# The impact of dysbiosis in oropharyngeal and gut microbiota on systemic inflammatory response and short-term prognosis in acute ischemic stroke with preceding infection

**DOI:** 10.3389/fmicb.2024.1432958

**Published:** 2024-08-22

**Authors:** Qiuxing He, Guoshun Li, Jiasheng Zhao, Huishan Zhu, Huanhao Mo, Zhanshi Xiong, Zhan Zhao, Jingyi Chen, Weimin Ning

**Affiliations:** ^1^Department of Neurology, Dongguan Hospital of Guangzhou University of Chinese Medicine, Dongguan, China; ^2^South China Research Center for Acupuncture and Moxibustion, Medical College of Acu-Moxi and Rehabilitation, Guangzhou University of Chinese Medicine, Guangzhou, China

**Keywords:** acute ischemic stroke, preceding infection, upper respiratory tract infections, functional outcome, oropharyngeal microbiota, gut microbiota

## Abstract

**Background:**

Stroke is a devastating disease and ranks as the second leading cause of death and disability globally. Several studies have shown that preceding infection (PI) of upper respiratory tract are strongly associated with acute ischemic stroke (AIS). However, the clinical implications and underlying pathological mechanisms remain unclear.

**Methods:**

In this study, 16S rRNA gene sequencing was employed to compare the structural characteristics of oropharyngeal and gut microbiota in AIS patients with or without PI and normal controls (NCs; 30 cases each), and systemic inflammatory markers were detected to explore the relationship between upper respiratory tract infections (URTIs) and subsequent stroke severity and functional outcome and the potential mechanism.

**Results:**

We found that patients with AIS-PI exhibited elevated serum WBC, NE, CRP, and Hcy levels, as well as a higher 90-day mRS score. Oropharyngeal and gut microbiota analysis showed that AIS and AIS-PI patients exhibited increased microbial richness in sequence. Principal coordinate analysis of the microbiota demonstrated significant differences in microbiota composition among the three groups. In AIS-PI patients, *Megamonas*, *Megasphaera*, *Ruminococcaceae UCG 004*, *Rothia*, and *Streptococcus* were significantly enriched in the gut. Opportunistic pathogens, including *Thermus*, *uncultured Veillonella* sp., and *Oribacterium sinu*, were found to be significantly enriched in the oropharynx. The dysregulated microbiota were positively correlated with systemic inflammatory markers, stroke severity, and poor prognosis. In contrast, short-chain fatty acid-producing bacteria *Eisenbergiella*, *bacterium NLAE*, *Fusicatenibacter*, *Ruminococcaceae*, and *Faecalibacterium* were enriched in NCs. Their abundances were negatively correlated with systemic inflammatory markers, stroke severity and poor prognosis.

**Conclusion:**

Our findings suggest that PIs of the upper respiratory tract may contribute to poor short-term functional outcome in AIS patients by causing disturbance of the oropharyngeal and gut microbiota and promoting elevated systemic inflammation levels.

## 1 Introduction

Stroke is a devastating disease and ranks as the second leading cause of death and disability globally. According to data released by the Global Burden of Disease Institute, in 2019, there were more than 122 million stroke cases worldwide, with an estimated global incidence rate of 81.9 to 110.76 per 100,000 people. The number of stroke deaths exceeded 6.55 million, and the estimated global mortality rate reached 39.08 to 46.77 per 100,000 people (Feigin et al., 2021). Acute ischemic stroke (AIS) accounts for more than 80% of all strokes (Virani et al., 2020). Currently, the treatment methods for AIS are very limited. The only evidence-based treatments available are recanalization therapies within a strict time window, such as intravenous thrombolysis and mechanical thrombectomy. However, most patients fail to receive timely treatment within the time window, which has a negative impact on prognosis (Saini et al., 2021). Consequently, controlling risk factors is crucial to reduce the incidence and severity of stroke and improve patient outcomes (Barber et al., 2001). Notably, in addition to the well-established risk factors, there are now indications that upper respiratory tract infections (URTIs) may represent an additional potential stroke risk.

A number of epidemiological studies conducted both domestically and internationally have shown that: 10–25% of IS patients have preceding infection (PI) of upper respiratory tract. Moreover, there is a significant increase in the risk of IS within 1–3 days following URTIs (Grau et al., 2010; Ohland et al., 2020). A prospective cohort study involving over 15,000 adults confirmed that infections may act as triggers for AIS, with the highest risk of stroke occurring 3–15 days after infection (Clayton et al., 2008; Elkind et al., 2011; Cowan et al., 2016). Therefore, the occurrence of URTIs is considered to be an important “trigger point” for AIS (Elkind et al., 2020). AIS patients with PIs may constitute a distinct subset with varied responses to acute ischemic injury compared to those without such antecedents, potentially impacting functional outcomes and prognosis (Heikinheimo et al., 2013; Elkind et al., 2020). Studies have demonstrated that AIS-PI patients exhibited unfavorable short-term outcomes (Grabska et al., 2011; Heikinheimo et al., 2013). Additionally, AIS patients with concurrent COVID-19 infection tend to be younger, exhibit more severe strokes, and display a higher incidence of large vessel occlusion compared to non-infected AIS patients (Stein et al., 2021). However, the precise mechanism by which PIs contribute to stroke onset and progression remains elusive. Proposed mechanisms may involve disruptions to the gut microbiota (Zhu et al., 2021), systemic release of cytokines and other inflammatory mediators leading to a prothrombotic state, and inflammation-mediated endothelial damage (Stein et al., 2021).

Microbiota dysbiosis plays a pivotal role in the pathogenesis of AIS. The gastrointestinal tract and oropharynx are primary locations where commensal microorganisms are distributed throughout the human body. In recent years, numerous studies have demonstrated not only the close association between gut microbiota and stroke risk factors such as hypertension (Adnan et al., 2017), diabetes (Forslund et al., 2015) and hyperlipidemia (Fu et al., 2015), but also its direct impact on the occurrence, progression, and prognosis of stroke (Pluta et al., 2021). The gut microbiota has emerged as a key regulator of brain function. Dysbiosis in the gut microbiota may exacerbate neurological damage in AIS, thereby contributing to the escalation of the inflammatory cascade in the nervous system (Singh et al., 2016). Studies have found that patients with IS or transient ischemic attack exhibit increased levels of pathogenic bacteria and reduced levels of probiotics in the gut compared to healthy controls. Additionally, the severity of the disease correlates with the degree of dysbiosis (Yin et al., 2015).

The oropharyngeal microbiome ranks among the most diverse microbial communities in the human body and plays a critical role in maintaining health and contributing to disease processes (Morris et al., 2018). Local conditions such as periodontal disease and respiratory tract infections can disrupt microbial homeostasis in the oropharynx (Gao et al., 2018). Nevertheless, the oropharynx is a highly vascularized area, which allows bacteria to translocate across the epithelial mucosa, leading to bacteremia (Kumar, 2017), and may also facilitate colonization shifts, disturbing the gut microbiota, thereby triggering disseminated infections, injuries, and inflammation (Kumar, 2017; Kitamoto et al., 2020). Recent investigations have revealed that respiratory infections can alter blood–brain barrier permeability, inciting inflammation within the central nervous system (Bohmwald et al., 2021). Additionally, studies suggest that the oral saliva microbiota of IS patients and individuals at high risk for IS exhibit considerable diversity, with specific bacterial profiles holding predictive value for IS severity and prognosis (Sun et al., 2023). Pulmonary infections have been shown to exacerbate IS outcomes, and post-IS brain injury can adversely affect the gut and lungs, indicating bidirectional feedback among these organs and the brain (Xie et al., 2024). Overall, these findings underscore the potential of oropharyngeal and gut microbiota as biomarkers for diagnosing and managing AIS. However, whether URTIs promote the occurrence, development, and adverse outcomes of AIS by perturbing the balance of oropharyngeal and gut microbiota remains unclear.

In this study, we utilized 16S rRNA gene sequencing to compare the structures of oropharyngeal and gut microbiota among AIS patients with or without PIs of the upper respiratory tract, along with normal controls (NCs). Furthermore, we evaluated systemic inflammatory markers to explore the association and potential mechanism linking URTIs with subsequent stroke severity and functional outcomes.

## 2 Materials and methods

### 2.1 Study participants

Between February 2021 and March 2022, we enrolled a total of 90 individuals from the Neurology Department at Dongguan Hospital of Traditional Chinese Medicine. The participants included NCs (*n* = 30), AIS patients (*n* = 30), AIS with PIs of the upper respiratory tract (AIS-PI; *n* = 30). Diagnosis of AIS was conducted by experienced neurologists following the guidelines outlined in the “Chinese guidelines for diagnosis and treatment of acute ischemic stroke 2018” (Neurology CSO and Society CS, 2018). PIs of the upper respiratory tract were defined as the presence of two or more typical local infection symptoms of upper respiratory infections, or at least one typical symptom accompanied by fever within 2 weeks before AIS onset (Grau et al., 1998). Fever was defined as a body temperature ≥ 38.0°C. NCs, matched for age and sex to the patients and lacking any history of cardiovascular disease or active infection, were voluntarily recruited from the same institution. To maintain the study’s integrity, we excluded participants meeting any of the following criteria: (Feigin et al., 2021) presence of malignant tumors, immune-mediated inflammatory disease, or digestive system diseases (such as chronic constipation, chronic diarrhea, and organic diseases), severe mental illnesses, etc.; (Virani et al., 2020) recent use of probiotics or antibiotics within 3 months prior to inclusion; (Saini et al., 2021) history of gastrointestinal surgery; (Barber et al., 2001) inability to cooperate with the study due to other reasons. This study received approval from the Ethics Committee of Dongguan Hospital of Traditional Chinese Medicine [Approval No. PJ (2021) NO. 33], and written informed consent was obtained from all participants and their legal guardians.

### 2.2 Clinical data and sample collection

Participants’ demographic information and medical histories were collected in a face-to-face interview within 24 h of admission. The degree of neurological deficit was assessed by two neurologists (Chen J and Zhao Z) using the National Institutes of Health Stroke Scale (NIHSS). Functional outcomes were evaluated at the follow-up points of 3 months and 1 year post-stroke using the modified Rankin Scale (mRS) score via telephone interviews.

Throat swabs and fasting blood samples were obtained within 24 h of admission, while fecal samples were collected within 48 h. Peripheral venous blood samples were collected drawn after an overnight fast and analyzed at the hospital’s central laboratory using automatic routine blood analysis. Throat swabs were procured by a physician after swabbing bilateral velopharyngeal arch and posterior pharyngeal wall with sterile disposable sampling cotton swabs, following gargling with clean water for 3 to 5 times. Sterile fecal containers, equipped with a spoon, were utilized for fecal samples collection, with each sample weighing between 5 and 10 grams. Throat swab and fresh fecal samples were promptly stored at −80°C until analysis.

### 2.3 DNA extraction and analysis of oropharyngeal and gut microbiota

Fecal and throat swab DNA were extracted utilizing the Feces Genomic DNA Purification Kit and Bacterial DNA Extraction Mini Kit, respectively, following the manufacture’s instructions. DNA integrity and purity were assessed via 1% agarose gel electrophoresis, while the purity and concentration of DNA were determined using NanoDrop TMOne. Subsequently, DNA fragmentation, end repair, adapter ligation and PCR amplification were employed to construct the DNA libraries. The quality of these libraries was evaluated using an Agilent 2100 Bioanalyzer. Libraries passing quality control standards were sequenced using the Illumina Nova 6000 platform.

The original sequencing were filtered and spliced to obtain clean data. Subsequently, the data were clustered to generate Operational Taxonomic Units (OTUs). Species annotation information was acquired by aligning with the representative sequences of selected OTUs from the Silva (16S) database. Furthermore, relevant flora information of each sample at various taxonomic levels (phylum, class, order, family, genus, and species) was obtained. Alpha diversity was assessed using usearch-alph_div v10.0.240 to describe the abundance and diversity of microbiota, encompassing richness, Chao1, Shannon_2, and Simpson indexes. Beta diversity was employed to further analyze the differences in community structure among different samples, utilizing principal coordinate analysis (PCoA) for analysis and visualization. To identify significantly different taxa between groups, Linear Discriminant Analysis (LDA) coupled with effect size (LEfSe) was implemented. Only values with an absolute logarithmic LDA score > 3 and *p* < 0.05 were deemed statistically significant. Lastly, associations between the relative abundance of microbiota and stroke severity, functional outcomes and biochemical parameters were detected by Spearman correlation analysis.

### 2.4 Statistical analysis

Statistical analyses were conducted utilizing GraphPad Prism software (version 9.0.0) and Origin software (version 2021). Continuous variables were presented as median values with interquartile range (medians, IQR), contingent upon the outcome of the Shapiro–Wilk test for normality. Group comparisons for continuous variables were conducted using either the Mann–Whitney test or the Kruskal-Wallis test, with subsequent Dunn’s post-hoc tests applied where appropriate. For comparisons of categorical variables, the Chi-square test or Fisher’s exact test was employed. Bivariate correlations were examined using Spearman correlation analysis. Statistical significance was defined as a two-tailed *p*-value < 0.05.

## 3 Results

### 3.1 Host clinical characteristics associated with stroke severity and poor functional outcome

The basic clinical information of the study, encompassing 30 AIS patients, 30 AIS-PI patients, and 30 normal controls, is presented in Table 1 and Supplementary Table 1. Parameters such as age, gender, smoking, drinking habits, and medical history of hypertension, diabetes mellitus, and hyperlipidemia were found to be comparable among the three groups (*p* > 0.05), indicating that baseline characteristics were similar across the cohorts.

**Table 1:** Characteristics of the study participants.

Characteristic^a^	NC (*n* = 30)	AIS patients (*n* = 30)	AIS-PI patients (*n* = 30)	*p*-value
**Demographic and clinical features**				
Mean age, median (IQR), y	58.50 (51.75–67.00)	60.00 (47.75–68.25)	59.50 (51.00–74.50)	0.5906
Sex, Male (*n*, %)	21, 70.00	18, 60.00	20, 66.67	0.7086
Smoker (*n*, %)	8, 26.67	10, 33.33	11, 36.67	0.7004
Drinking (*n*, %)	4, 13.33	9, 30.00	9, 30.00	0.262
**Medical history**				
Hypertension (*n*, %)	-	21, 70.00	23, 76.67	0.7710
Diabetes mellitus (*n*, %)	-	8, 26.67	10, 33.33	0.7787
Hyperlipidemia (*n*, %)	-	7, 23.33	9, 30.00	0.7710
NIHSS before, median (IQR)	-	3.00 (2.00–5.25)	4.00 (2.00–7.25)	0.3492
NIHSS after, median (IQR)	-	1.50 (0.75–3.25)	3.00 (1.00–6.00)	0.1458
mRS after, median (IQR)	-	1.00 (0.00–1.25)	1.00 (0.00–3.00)	0.6308
**Laboratory data, median (IQR)**				
WBC count, 10^9^/L	7.40 (7.15–7.68)	6.88 (5.81–8.14)	8.67 (7.04–11.13)	0.004
NE count, 10^9^/L	4.13 (4.13–4.65)	4.66 (4.07–5.42)	6.05 (4.29–8.56)	0.010
CRP level, mg/L	1.51 (1.12–3.51)	1.86 (1.13–4.62)	3.58 (1.49–12.32)	0.0527
Hcy level, μmol/L	11.00 (8.75–12.95)	13.55 (10.00–18.53)	14.15 (9.88–20.10)	0.0169
**Follow-up, median (IQR)**				
mRS 90d	-	0.00 (0.00–2.00)	2.00 (0.00–3.00)	0.0198
mRS 1y	-	0.00 (0.00–1.25)	1.00 (0.00–3.00)	0.0948

NC, normal controls; AIS patients, acute ischemic stroke patients; AIS-PI patients, acute ischemic stroke patients with preceding infections; IQR, interquartile range; NIHSS, National Institutes of Health Stroke Scale; mRS, modified Rankin Scale; WBC, white blood cell; NE, Neutrophil; CRP, C-reactive protein; Hcy, homocysteine.

a Data are reported as number (%) for categorical variables and median (IQR) for continuous variables.

**Figure 1:**
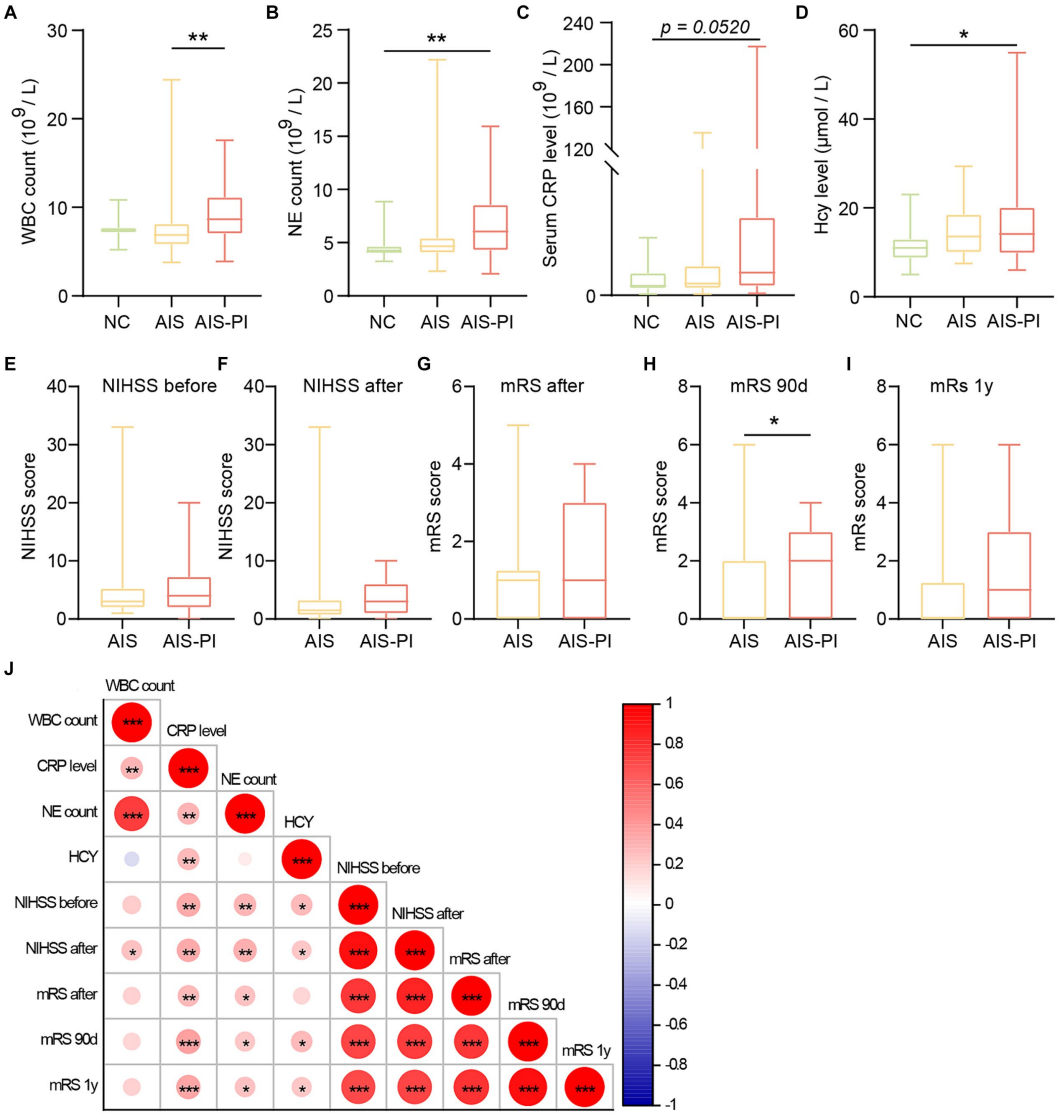
Systemic inflammation and poor functional outcome in AIS-PI patients. **(A–D)** Comparison of systemic inflammatory biomarker levels [WBC count **(A)**, NE count **(B)**, CRP level **(C)**, Hcy level **(D)**] in NCs, AIS patients and AIS-PI patients. NIHSS score before **(E)** and after **(F)** treatment in AIS and AIS-PI patients. The mRS score after treatment **(G)** and at 90d **(H)** and 1y **(I)** follow-up in AIS and AIS-PI patients. Heatmap of the Spearman correlation analysis between systemic inflammatory biomarkers and stroke severity and functional outcome **(J)**. Results are expressed as median and interquartile range. *p* < 0.05 indicates statistical significance (*p < 0.05, **p < 0.01, ***p < 0.0001).

As the AIS-PI group had experienced URTIs 14 days prior to the stroke, we examined the main markers associated with inflammation in blood tests. Analysis of the blood tests revealed that AIS-PI patients exhibited higher plasma white blood cell (WBC) counts than AIS patients (Figure 1A). Additionally, AIS-PI patients showed higher plasma neutrophil (NE) counts and homocysteine (Hcy) levels when compared to the NC group (Figures 1B,D). While C-reactive protein (CRP) levels were also elevated in the AIS-PI group compared to the NC group, but not significantly (*p* = 0.0520; Figure 1C). CRP, an inflammatory marker produced by the liver, and elevated levels of Hcy typically indicate tissue damage from infection (Yao et al., 2017). These findings collectively suggest a systemic inflammatory response following URTIs.

However, there were no significant differences in NIHSS scores before and after treatment between the AIS and AIS-PI groups (Figures 1E,F). At the 90-day follow-up, the AIS-PI patients displayed elevated mRS scores, indicative a poorer functional outcome (Figures 1G–I). Further investigation into the correlations between clinical characteristics and stroke severity and functional outcome revealed positive associations among plasma inflammatory markers. Moreover, plasma CRP, Hcy, and NE counts exhibited significant positive correlations with stroke severity and poor functional outcomes, including NIHSS before and after treatment, mRS after treatment, mRS at 90 days, and mRS at 1 year (Figure 1J).

### 3.2 Alterations of gut microbiota in the AIS patients, AIS-PI patients and NCs

**Figure 2:**
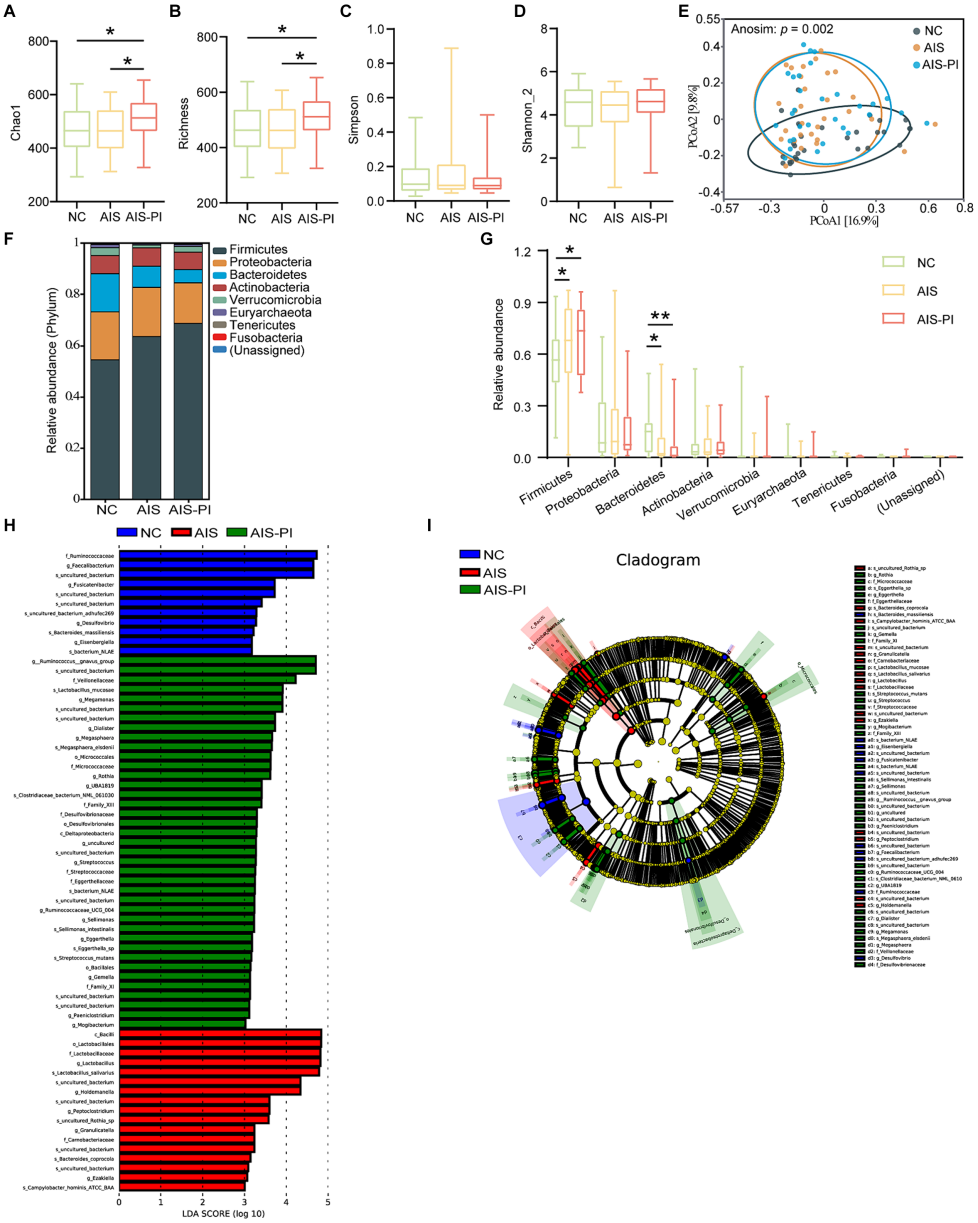
Comparison of gut microbiota in NCs, AIS patients and AIS-PI patients. **(A–D)** α-diversity was evaluated by Chao1, Richness, Simpson, and Shannon_2 index. **(E)** β-diversity from the Bray-Curtis distance was calculated by PCoA based on the Anosim analysis (*p* = 0.002, AIS vs. NCs: *p* = 0.003, AIS-PI vs. NCs: *p* = 0.002, AIS-PI vs. AIS: *p* = 0.052). **(F,G)** The top 10 relative abundances of gut microbiota at the phylum level. Significantly discriminant taxa (from phylum to species) in three groups identified by linear discriminant analysis (LDA) scores and LDA effect size (LEfSe) analysis (LDA score threshold: ≥ 3.0). Histogram **(H)** and cladogram **(I)** of the LEfSe analysis of the gut microbiota in NCs (blue), AIS patients (red) and AIS-PI patients (green). Results are expressed as median and interquartile range. *p* < 0.05 indicates statistical significance (*p < 0.05, **p < 0.01).

To investigate potential changes in the gut microbiota of AIS patients and AIS-PI patients, we conducted a comparison of fecal microbiome diversity and composition among the three groups using 16S metagenomic sequencing. Our analysis revealed significant differences in α-diversity indices, particularly Chao 1 and Richness, which were notably higher in AIS-PI patients compared to both NCs and AIS patients, while no significant differences were observed in Simpson and Shannon_2 indices (Figures 2A–D). These findings suggest a significantly greater richness of gut microbiota in AIS-PI patients. To further explore whether the microbial community composition differed between AIS-PI patients, AIS patients, and NCs, Principal Coordinate Analysis (PCoA) plots based on Bray–Curtis distance were generated. The results unveiled noticeable inter-individual distinctions in AIS and AIS-PI patients compared to NCs (Figure 2E), suggesting a dysbiosis of gut flora in AIS and AIS-PI patients.

Further analysis revealed distinct microbial profiles in AIS patients and AIS-PI patients compared to NCs at various taxonomic levels. At the phylum level, *Firmicutes*, *Bacteroidetes*, *Proteobacteria*, and *Actinobacteria* were the dominant phyla in all three groups, collectively constituting more than 90.0% of the total bacterial flora (Figure 2F). In comparison to NCs, the *Firmicutes* phylum was significantly more abundant in AIS and AIS-PI patients, while the *Bacteroidetes* phylum was relatively less abundant (Figure 2G). Furthermore, LEfSe analysis and LDA score were used to identify microbial species with significant differences from phylum to genus among the three groups. Several opportunistic pathogens, including the genus *Megamonas*, the species *Lactobacillus mucosae*, the genus *Megasphaera* and its corresponding species *Megasphaera elsdenii*, genus *Dialister*, the order *Micrococcales* and its corresponding family *Micrococcaceae* and genus *Rothia*, the family *Streptococcaceae* and its corresponding genus *Streptococcus* and the species *Streptococcus mutans*, the genus *Ruminococcaceae UCG 004*, the genus *Sellimonas* and its corresponding species *Sellimonas intestinalis*, the family *Eggerthellaceae* and its corresponding genus *Eggerthella* and species *Eggerthella* sp., the genus *Paeniclostridium* and the genus *Mogibacterium* were enriched in the AIS-PI patients, while the species *Campylobacter hominis ATCC BAA* and *Bacteroides coprocola* were more abundant in the AIS patients. NCs exhibited a higher abundance of short-chain fatty acids (SCFAs)-producing bacteria, including the genus *Eisenbergiella* and its corresponding species *bacterium NLAE*, genus *Fusicatenibacter*, family *Ruminococcaceae* and its corresponding genus *Faecalibacterium*, and species *Bacteroides massiliensis* (Figures 2H,I).

### 3.3 Gut microbiota dysbiosis correlated with systemic inflammatory and stroke severity and poor functional outcome

**Figure 3:**
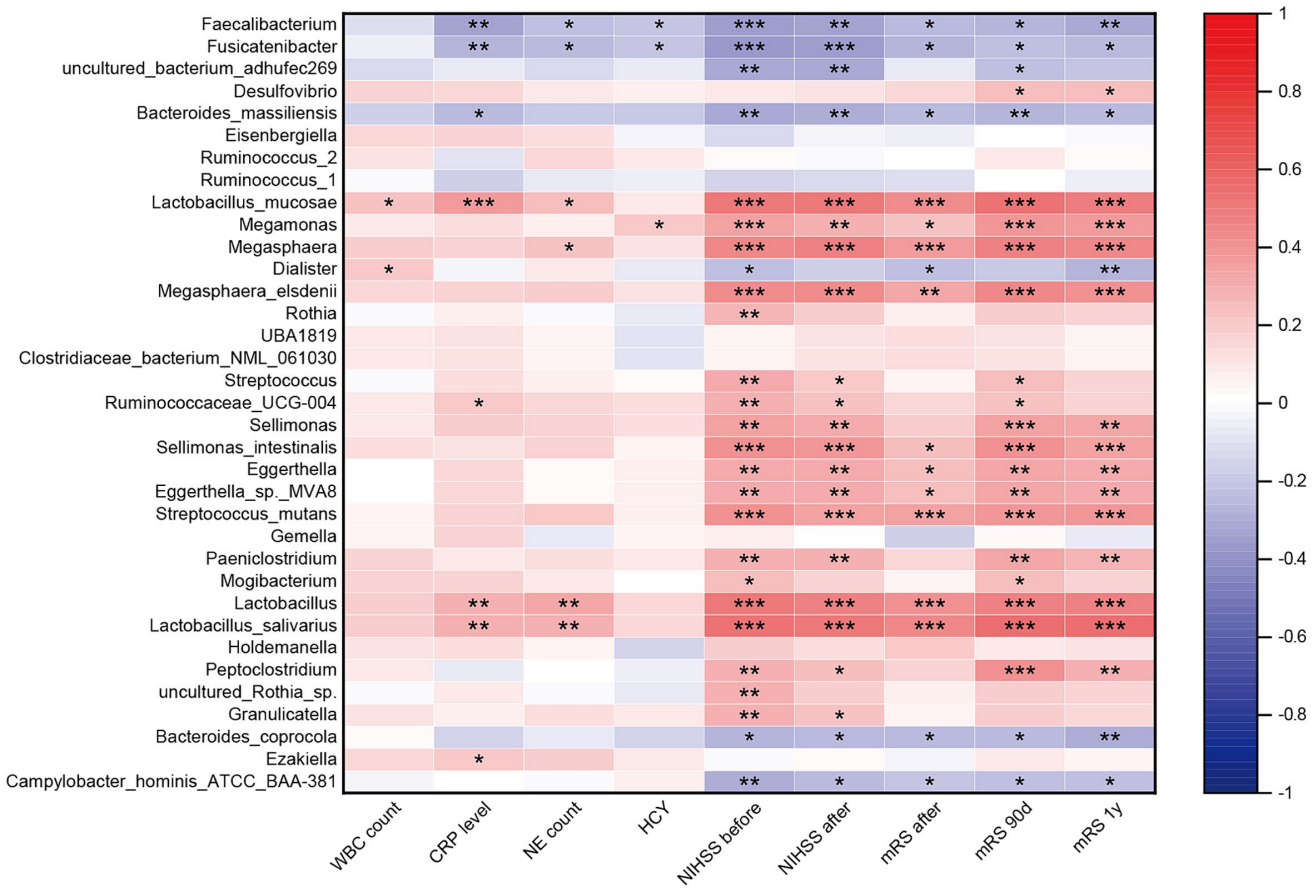
Correlation between the gut microbiota dysbiosis and systemic inflammatory and stroke severity and functional outcome. Heatmap of the Spearman correlation analysis between the dysbiosis of the gut microbiota and systemic inflammatory, stroke severity and functional outcome. *p* < 0.05 indicates statistical significance (*p < 0.05, **p < 0.01, ***p < 0.0001).

To investigate the association between gut microbiota dysbiosis and systemic inflammatory, stroke severity and functional outcome in the three groups, we conducted correlation analysis using Spearman’s correlation. Our results revealed significant correlations between specific microbial taxa and these clinical parameters. SCFAs-producing bacteria such as *Fusicatenibacter* and *Faecalibacterium*, and *Bacteroides massiliensis*, exhibited negative correlations with systemic inflammatory, stroke severity, and poor functional outcome. Conversely, pathogenic bacterium including *Megamonas*, *Lactobacillus mucosae*, *Megasphaera*, and *Ruminococcaceae UCG 004* showed positive correlations with systemic inflammation, stroke severity, and poor functional outcome. Additionally, other pathogenic bacterium such as *Megasphaera elsdenii*, *Streptococcus*, *Streptococcus mutans*, *Sellimonas*, *Sellimonas intestinalis*, *Eggerthella*, *Eggerthella* sp., *Paeniclostridium*, and *Mogibacterium* displayed positive correlations specifically with stroke severity and poor functional outcome. Notably, the species *Campylobacter hominis ATCC BAA* and *Bacteroides coprocola*, enriched in AIS-PI patients, exhibited negative correlations with stroke severity and poor functional outcome (Figure 3).

### 3.4 Alterations of oropharyngeal microbiota in the AIS patients, AIS-PI patients and NCs

**Figure 4:**
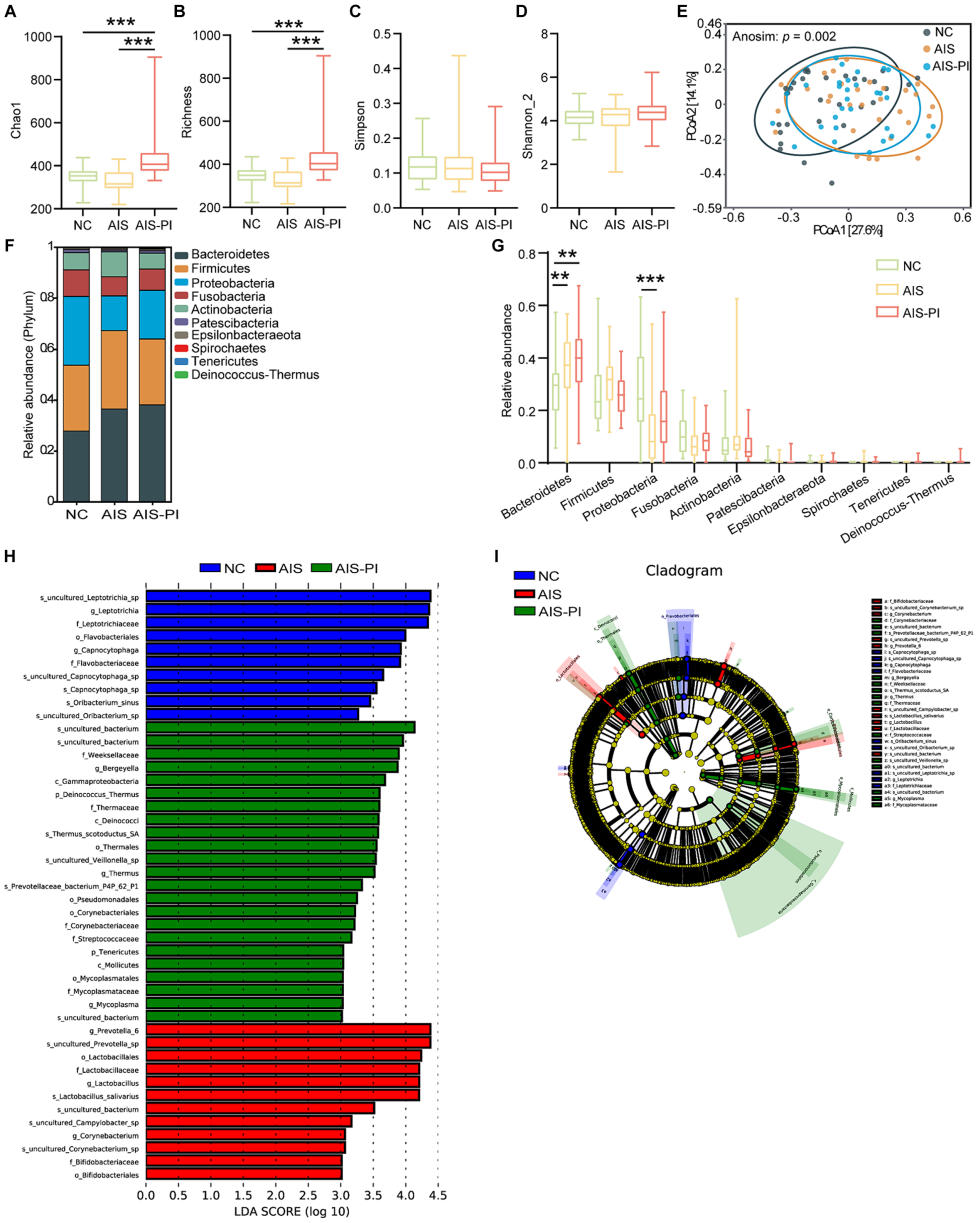
Comparison of oropharyngeal microbiota in NCs, AIS patients and AIS-PI patients. **(A–D)** α-diversity was evaluated by Chao1, Richness, Simpson, and Shannon_2 index. **(E)** β-diversity from the Bray-Curtis distance was calculated by PCoA based on the Anosim analysis (*p* = 0.003, AIS vs. NCs: *p* = 0.006, AIS-PI vs. NCs: *p* = 0.003, AIS-PI vs. AIS: *p* = 0.692). **(F,G)** The top 10 relative abundances of oropharyngeal microbiota at the phylum level. Significantly discriminant taxa (from phylum to species) in three groups identified by linear discriminant analysis (LDA) scores and LDA effect size (LEfSe) analysis (LDA score threshold: ≥ 3.0). Histogram **(H)** and cladogram **(I)** of the LEfSe analysis of the oropharyngeal microbiota in NCs (blue), AIS patients (red) and AIS-PI patients (green). Results are expressed as median and interquartile range. *p* < 0.05 indicates statistical significance (*p < 0.05, **p < 0.01).

The colonization of the intestine by oropharyngeal microbiota has been linked to several diseases. To further investigate whether oropharyngeal microbial dysbiosis is present in AIS-PI patients, we conducted a series of analyses. Firstly, α-diversity and β-diversity analyses were performed to assess potential differences in oropharyngeal microbiota composition among the three groups. Our results revealed significant differences in α-diversity indices, particularly Chao 1 and Richness, which were notably higher in AIS-PI patients compared to both NCs and AIS patients. However, no significant differences were observed in the Simpson and Shannon_2 indices (Figures 4A–D). These findings suggest that oropharyngeal microbiota richness was significantly elevated in AIS-PI patients, consistent with the gut microbial findings. Furthermore, significant disparities were observed in oropharyngeal microbiota communities in AIS and AIS-PI patients compared to NCs, as indicated by the results of PCoA based on Bray-Curtis distance and Anosim test (Figure 4E).

We further evaluated the composition of the oropharyngeal microbiota community among the three groups. At the phylum level, *Bacteroidetes*, *Firmicutes*, *Proteobacteria*, *Fusobacteria*, and *Actinobacteria* were the dominant phyla in all three groups, accounting for more than 90.0% of the total bacterial flora (Figure 4F). In comparison to the NCs, the *Bacteroidetes* phylum was significantly more abundant in AIS and AIS-PI patients, while the *Proteobacteria* phylum was relatively less abundant in AIS patients (Figure 4G). Further analysis using LEfSe and LDA score showed that the family *Streptococcaceae and Corynebacteriaceae*, the *uncultured bacterium* of *Rothia* genus, the phylum *Deinococcus Thermus* and its corresponding class *Deinococci*, order *Thermales*, family *Thermaceae*, genus *Thermus* and species *Thermus scotoductus SA*, the species *uncultured Veillonella* sp., and the genus *Mycoplasma* were enriched in the AIS-PI patients, while the family *Leptotrichiaceae* and its corresponding genus *Leptotrichia* and species *uncultured_Leptotrichia_*sp., and species *Oribacterium sinus* were less abundant (Figures 4H,I). These findings provide insights into the specific microbial taxa associated with oropharyngeal dysbiosis in AIS-PI patients, shedding light on potential microbial biomarkers and therapeutic targets for further investigation.

### 3.5 Oropharyngeal microbiota dysbiosis correlated with systemic inflammatory and stroke severity and poor functional outcome

**Figure 5:**
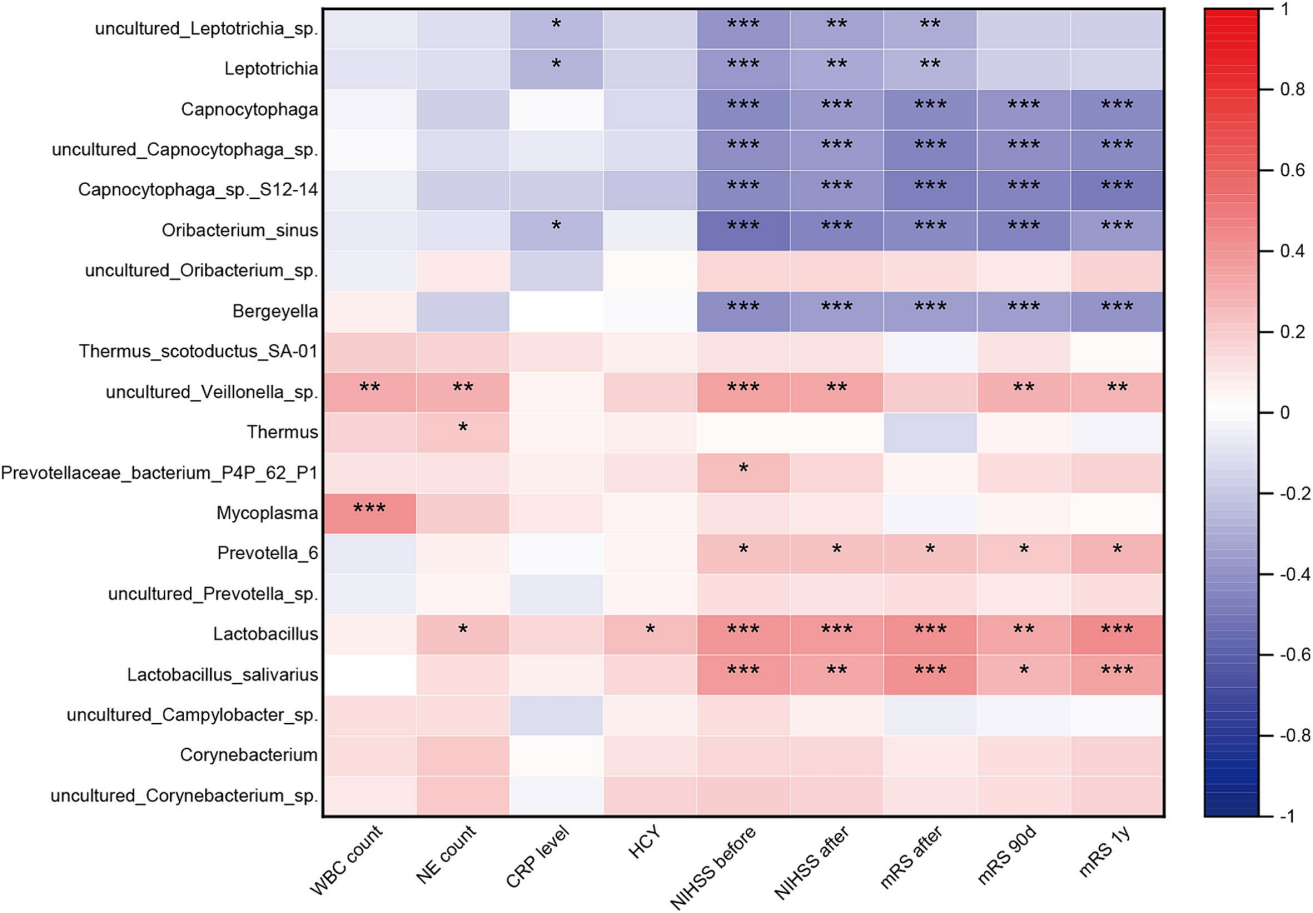
Correlation between the oropharyngeal microbiota dysbiosis and systemic inflammatory and stroke severity and functional outcome. Heatmap of the Spearman correlation analysis between the dysbiosis of the oropharyngeal microbiota and systemic inflammatory, stroke severity and functional outcome. *p* < 0.05 indicates statistical significance (*p < 0.05, **p < 0.01, ***p < 0.0001).

Spearman correlation analyses unveiled significant associations between specific microbial taxa and clinical parameters. Inflammation-associated bacteria *Thermus* and *Mycoplasma* exhibited a positive correlation with the inflammatory biomarker, while *Veillonella* sp. displayed a positive correlation with systemic inflammation, stroke severity, and poor functional outcome. Conversely, the *Leptotrichia* genus, *Leptotrichia* sp., and *Oribacterium sinus* demonstrated a negative correlation with CRP levels, stroke severity, and poor functional outcome (Figure 5).

### 3.6 Oropharyngeal microbiota dysbiosis correlated with gut microbiota dysbiosis

**Figure 6:**
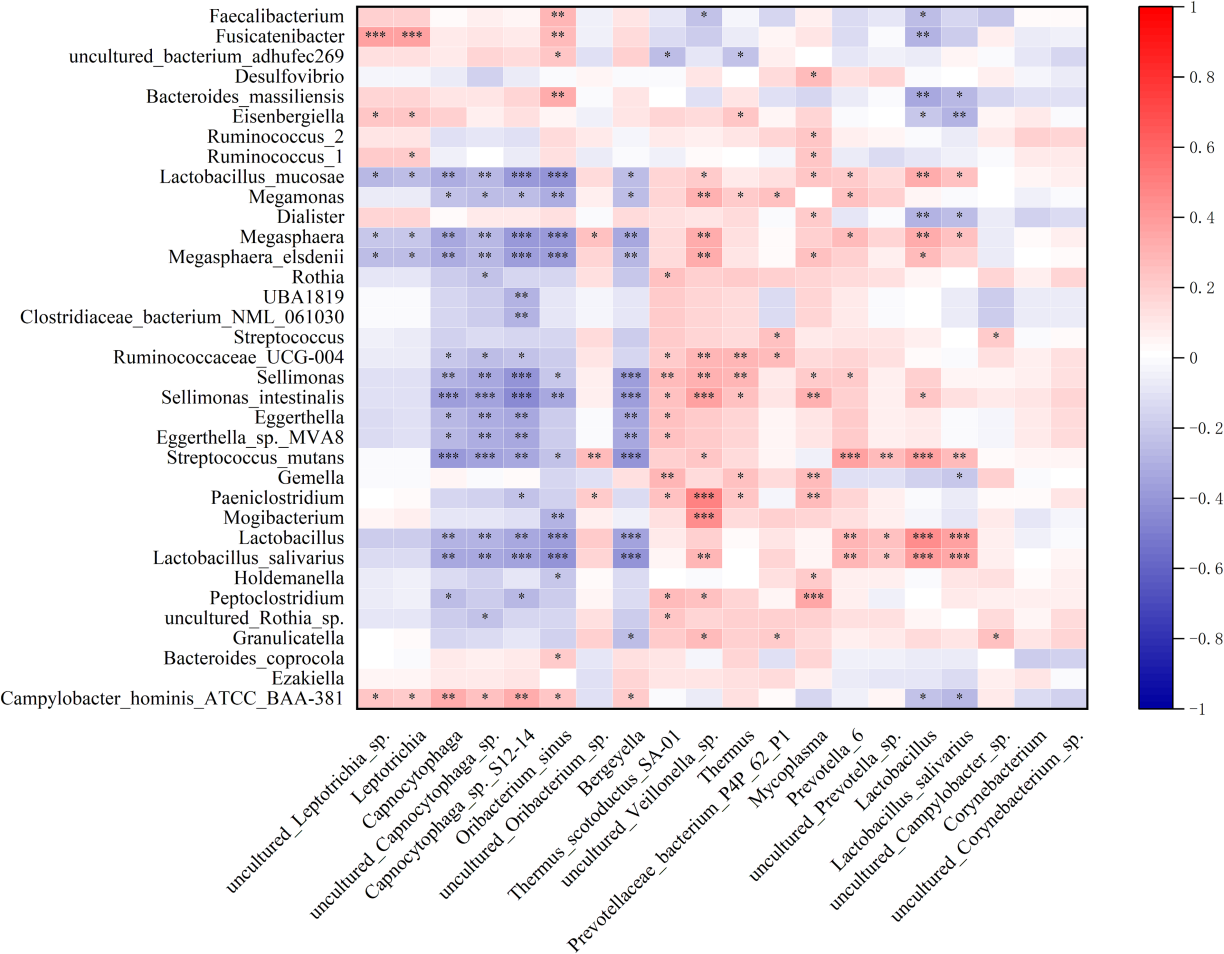
Correlation between the relative abundance of the differential oropharyngeal microbiome and gut microbiome. Heatmap of the Spearman correlation analysis between the relative abundance of the differential oropharyngeal microbiome and gut microbiome. *p* < 0.05 indicates statistical significance (*p < 0.05, **p < 0.01, ***p < 0.0001).

Spearman correlation analysis revealed a significant association between the dysbiotic microbiota in the oropharynx and the dysbiotic microbiota in the gut (Figure 6).

## 4 Discussion

Increasing evidence suggests that the interaction between gut microbiota and the immune system is crucial for the onset and severity of stroke (Zheng et al., 2022). While previous studies primarily focused on microbial composition differences between healthy controls and AIS patients, our study is the first to analyze the interplay between oropharyngeal and gut microbiota across normal controls, AIS patients, and AIS-PI patients. Our findings show that AIS-PI patients exhibit elevated systemic inflammation levels and poor short-term functional outcomes, with significant variations in the composition of gut and oropharyngeal microbiota among the three groups. Importantly, microbial imbalance is linked to systemic inflammation levels, NHISS scores, and mRS scores.

Previous research has demonstrated that PI is independently associated with adverse short-term outcomes (Heikinheimo et al., 2013). This study consistently demonstrated that AIS-PI patients had worse 90-day outcomes despite no significant increase in the NIHSS score when compared to AIS patients. Inflammation plays a crucial role in the pathogenesis of AIS (Lindsberg and Grau, 2003; Yamashiro et al., 2017) and correlates with stroke severity (Zheng et al., 2022). Our findings indicate that AIS-PI patients had elevated levels of serum WBC, NE, CRP, and Hcy, suggesting an active of systemic inflammatory response. Recent studies have indicated that elevated acute inflammatory markers, such as NE, arising from infection can exacerbate secondary inflammation post-AIS (Oh and Parikh, 2022), which might trigger further damage in the ischemic penumbra (Heiss, 2012). CRP is a significant inflammatory marker that has been shown to be elevated in AIS patients, correlating positively with more severe stroke conditions, higher mortality, and disability rates (Luo et al., 2012). Furthermore, early elevation of CRP and Hcy levels in AIS patients has been suggested as an independent prognostic indicator (Wang et al., 2019, 2023; Jia et al., 2024). Experimental animal studies have demonstrated that high Hcy levels can activate astrocytes and induce neurotoxicity in the brain following IS via the JAK2/STAT3 signaling pathway (Chen et al., 2017). Importantly, our study demonstrates a positive correlation between systemic inflammatory markers, including WBC count, NE count, CRP level, and Hcy level, with stroke severity and adverse functional prognosis. Therefore, URTIs may result in unfavorable short-term functional outcomes in AIS patients by inducing systemic inflammatory response.

We observed a significant increase in oropharyngeal and gut microbiota richness, as estimated by the Chao1 and Richness indices, in AIS-PI patients compared to NCs and AIS patients. β-diversity analysis results revealed significant differences in the composition and structure of oropharyngeal and gut microbiota in the AIS and AIS-PI patients compared with NCs.

Compared to NCs, both AIS and AIS-PI patients exhibit an increase in *Firmicutes* and a decrease in *Bacteroidetes* in the gut, with AIS-PI patients showing a greater magnitude of change. Previous studies have consistently indicated that the *Firmicutes* to *Bacteroidetes* (F/B) ratio is higher in non-minor stroke patients than in minor stroke patients (Gu et al., 2021). Experimental studies in animals have demonstrated that reducing the F/B ratio in elderly mice to levels similar to those observed in young mice can improve stroke prognosis (Spychala et al., 2018). LEfSe analysis revealed an enrichment of pathogenic microorganisms including *Megamonas*, *Megasphaera*, and *Ruminococcaceae UCG 004*, in the gut of AIS-PI patients. This enrichment positively correlated with systemic inflammation levels, stroke severity, and mRS scores. *Megamonas* is an inflammation-related bacterium (Balmant et al., 2023), and its abundance, along with HCY levels, has been shown to significantly increases in AIS and transient ischemic attack patients (Xu et al., 2018). *Rothia* is associated with URTIs (Kazachkov et al., 2018), *Streptococcus* is a major pathogen for dental caries and pneumonia (Bloch et al., 2024), while *Sellimonas* is linked to enteritis (Yan et al., 2022). This study found that these pathogenic microorganisms are enriched in the gut of AIS-PI patients, with their abundance positively correlating with stroke severity and mRS scores. This may be attributed to increased systemic inflammation levels and microbial translocation due to PI. Conversely, SCFAs-producing bacteria, including *Eisenbergiella*, *bacterium NLAE*, *Fusicatenibacter*, *Ruminococcaceae*, and *Faecalibacterium* are more abundant in NCs. These beneficial bacteria are significantly inversely correlated with systemic inflammation, stroke severity, and adverse prognosis. SCFAs, including acetic acid, propionic acid, and butyric acid, are vital bacterial metabolic products that enhance energy metabolism and intestinal barrier integrity (Kasubuchi et al., 2015). Previous research has provided compelling evidence that microbial communities producing butyrate can counteract systemic inflammation (van den Munckhof et al., 2018). Furthermore, there is evidence that SCFAs from microbial sources can modulate post-stroke recovery by influencing systemic and brain-resident immune cells (Sadler et al., 2020).

Inflammation plays a pivotal role in the pathogenesis of URTIs and AIS. The enrichment of specific microorganisms in the oropharynx may significantly contribute to the development of both conditions by eliciting a shared host inflammatory response. LEfSe analysis revealed an enrichment of *Streptococcaceae*, *Corynebacteriaceae*, the *uncultured bacterium of Rothia genus*, *Thermus*, *uncultured Veillonella* sp., and *Oribacterium sinus* in the oropharynx of AIS-PI patients. *Thermus*, a Gram-positive bacterium, has been associated with high-inflammatory colorectal cancer (Cao et al., 2023). Furthermore, this study identified a positive correlation between *Thermus* abundance and NE count. *Veillonella*, a Gram-negative obligate anaerobe present in the human oral cavity, typically enriches in an inflammatory context and can translocate to the intestines (Rojas-Tapias et al., 2022). Clinical studies have indicated that *Veillonella* and *Streptococcus* are enriched in stroke patients and those at high risk, with predictive value for IS severity and prognosis (Sun et al., 2023). Our research corroborated these findings, showing a positive correlation between the abundance of *uncultured Veillonella* sp. and systemic inflammation levels, stroke severity, and adverse outcomes. Conversely, we observed a higher abundance of certain species, such as *Leptotrichia* and *Oribacterium sinus*, in NCs, which may relate to oral health and hygiene practices. It has been demonstrated that these oral pathogens do not colonize the gastrointestinal tract of healthy individuals (Kitamoto et al., 2020).

Although microbial composition is site-specific, evidence suggests a degree of overlap and interaction between the oropharyngeal and gut microbiota (Chen et al., 2022). When pathogenic microorganisms in the oropharynx surpass a certain threshold, they can translocate to the gut, disrupting the colonization resistance of gut microbiota and leading to dysbiosis and inflammatory responses (Kitamoto et al., 2020). Notably, similar enrichments of pathogenic bacteria, including *Veillonellaceae*, *Streptococcaceae*, and *Rothia*, as well as their related species have been observed in both the oropharynx and gut of AIS-PI patients. Correlation analysis further indicated a significant association between the dysbiotic microbiota in the oropharynx and the dysbiotic microbiota in the gut. In AIS-PI patients, these dysbiotic microbial communities are positively correlated with inflammation levels, stroke symptom severity, and adverse functional prognosis. This evidence suggests that oropharyngeal microbial dysbiosis, resulting from antecedent URTIs, leads to gastrointestinal dysbiosis through microbial translocation, subsequently triggering systemic inflammatory responses (Lira-Junior and Bostrom, 2018) and contributing to poor short-term prognosis in AIS-PI patients. Furthermore, it is plausible that oropharyngeal and gut microbiota, along with their metabolites, may induce neuroinflammation via translocation or leakage through damaged mucosal and blood–brain barriers, a critical aspect of AIS pathogenesis (Morris et al., 2018).

## 5 Limitation

Our study has several limitations that should be considered. First, while we observed changes in the composition of oropharyngeal and gut microbiota in AIS-PI patients, and noted associations between dysbiotic microbiota and systemic inflammation, stroke severity, and adverse prognosis, we utilized 16S rRNA amplicon sequencing instead of shotgun metagenomic sequencing. This choice limited our ability to identify specific bacteria at the species level. Second, we collected oropharyngeal and fecal microbiota as well as serum samples, at a single time point, restricting our capacity to observe dynamic changes in inflammation levels and microbiota, as well as their real-time interactions with stroke severity and functional outcomes. Lastly, oral health and hygiene habits may also influence the oropharyngeal microbiota. In this study, we did not collect data on oral health, nor did we control for this variable in our analysis. Therefore, the observed differences in oropharyngeal microbiota may not be solely attributable to antecedent infections in all participants. It is possible that the enrichment of opportunistic pathogens in NCs may be a secondary consequence of poor oral health.

## 6 Conclusion

Our investigation revealed alterations in the oropharyngeal and gut microbiota of AIS-PI patients. These alterations are characterized predominantly by a diminished production of SCFA by beneficial bacteria and augmented presence of opportunistic pathogens. Furthermore, shifts in oropharyngeal and gut microbiota correlate with systemic inflammation levels, stroke severity, and adverse prognostic outcomes in AIS-PI patients. Consequently, it is postulated that antecedent URTIs may disrupt the oropharyngeal and fecal microbiota, thereby fostering escalated systemic inflammation and, consequently, an unfavorable short-term functional prognosis in AIS patients.

## Data Availability

The raw reads were deposited into the NCBI Sequence Read Archive (SRA) database (Bioproject: PRJNA1113203 and PRJNA1112525).
